# Th2-Oriented Immune Serum After SARS-CoV-2 Vaccination Does Not Enhance Infection *In Vitro*

**DOI:** 10.3389/fimmu.2022.882856

**Published:** 2022-04-08

**Authors:** Ning Luan, Tao Li, Yunfei Wang, Han Cao, Xingxiao Yin, Kangyang Lin, Cunbao Liu

**Affiliations:** ^1^ Institute of Medical Biology, Chinese Academy of Medical Sciences and Peking Union Medical College, Kunming, China; ^2^ Institute for Biological Product Control, National Institutes for Food and Drug Control and WHO Collaborating Center for Standardization and Evaluation of Biologicals, Beijing, China

**Keywords:** inactivated SARS-CoV-2 vaccine, spike protein subunit vaccine, aluminum adjuvant, Th2, antibody-dependent enhancement of infection

## Abstract

The relatively lower protection rate of the alum-adjuvanted inactivated severe acute respiratory syndrome coronavirus 2 (SARS-CoV-2) vaccines reminds us of the antibody-dependent enhancement (ADE) phenomenon observed in preclinical studies during the development of vaccines for Middle East respiratory syndrome coronavirus (MERS-CoV) and severe acute respiratory syndrome coronavirus 1 (SARS-CoV-1). In this study, using the S1 segment of the SARS-CoV-2 spike protein or inactivated whole SARS-CoV-2 virus as an antigen and aluminum as an adjuvant, the risk of ADE of infection with T helper 2 (Th2)-oriented immune serum from mice (N=6) and humans (N=5) was examined in immune cell lines, which show different expression patterns of Fc receptors. Neither the immune serum from alum-adjuvanted S1 subunit vaccines nor inactivated SARS-CoV-2 vaccination enhanced SARS-CoV-2 S pseudotyped virus infection in any of the tested cell lines *in vitro*. Because both of these Th2-oriented immune sera could block SARS-CoV-2 infection without ADE of infection, we speculate that the lower protection rate of the inactivated SARS-CoV-2 vaccine may be attributed to the lower neutralizing antibody titers induced or the pulmonary eosinophilic immunopathology accompanied by eosinophilic infiltration in the lungs upon virus exposure. Adjustment of the immunization schedule to elevate the neutralizing antibody levels and skew adjuvants toward Th1-oriented responses may be considered to increase the efficacies of both inactivated and spike protein-based subunit SARS-CoV-2 vaccines.

## Introduction

Previous experience on the development of coronavirus vaccines for severe acute respiratory syndrome coronavirus 1 (SARS-CoV-1) and Middle East respiratory syndrome virus (MERS-CoV) has revealed that the T helper 2 (Th2) response bias of these vaccines is accompanied by a pulmonary immune pathology characterized by eosinophil infiltration upon virus challenge, although the subunit vaccines based on either the spike protein or the inactivated vaccine combined with aluminum adjuvant exert certain protective effects on reducing the viral loads in animal models during a subsequent challenge (1–4). *In vitro* analyses show that serum obtained after the administration of these vaccines could enhance viral infection, mainly through the Fc receptors (FcRs) of immune cells (5, 6). Although these infections have been proven to be abortive, viral elimination is reportedly associated with the production of multiple antiviral and proinflammatory cytokines, which may result in vaccine-associated enhanced respiratory disease (VAERD) (5, 7–11). Correspondingly, adjuvants that promote Th1 response bias have been adopted to avoid or reduce the risk of VAERD and improve the protective effect of these vaccines in preclinical studies (12–14).

Coincidentally, all successful SARS-CoV-2 vaccines with a protection rate greater than 90% have exhibited Th1-cell-skewed responses of their spike protein antigens during preclinical and clinical studies (15–19). These vaccines include the mRNA vaccine BNT162b2 developed by BioNTech (Germany), mRNA-1273 developed by Moderna (USA), and the subunit vaccine NVX-CoV2373 developed by NOVAVAX (USA). While Th1-oriented responses are induced by the intracellular translation of spike proteins and the innate immunity mobilization ability for mRNA vaccines, NVX-CoV2373 relies on its Th1-cell-biasing adjuvant Matrix-M (20–22).

Aluminum, which induces typical Th2 response bias for subunit and inactivated vaccines, has recently been the only adjuvant in vaccines licensed worldwide for human use (23). Aluminum has been applied as the adjuvant in inactivated SARS-CoV-2 vaccines, which show a protection rate ranging from 50.7% to 83.5% according to recently published clinical phase III data (Clinical trials registration numbers: NCT04510207, NCT04456595, NCT04582344, and NCT04651790) (24–27). No enhanced respiratory disease (ERD) typical of an increased eosinophilic proinflammatory pulmonary response upon challenge has been detected in preclinical studies of these inactivated SARS-CoV-2 vaccines in either murine or nonhuman primate (NHP) pneumonia models (28–32). However, whether the relatively lower vaccine efficacy originates from lower induction of neutralization antibody production or the possibility of antibody-dependent enhancement (ADE) of infection caused by Th2-oriented serum and a subsequent pulmonary immune pathology remains to be determined (28–30).

S1 is the coronavirus spike protein segment that contains the N-terminal segment and the receptor-binding domain (RBD) responsible for viral attachment to host cells and is thus widely considered a potential coronavirus vaccine target (4, 33–35). When adjuvanted with alum, serum obtained after SARS-CoV-1 S1 subunit vaccination reportedly induces ADE of infection similar to that observed with serum obtained after vaccination with inactivated whole SARS-CoV-1 viruses (5). Considering several IgG1 subtype monoclonal antibodies targeting SARS-CoV-2 spike protein have been reported to induced ADE of infection *in vitro* recently, the risk of Th-2 oriented immune serum after SARS-CoV-2 vaccination that containing polyclonal antibodies targeting the spike protein to enhance virus infection is to be assessed (36–38). In this study, using the S1 segment of SARS-CoV-2 and inactivated whole SARS-CoV-2 virus as antigen and aluminum as an adjuvant, we studied the risk of ADE of infection with Th2-oriented immune serum from mice and humans in immune cell lines expressing different patterns of FcRs. We aimed to provide helpful clues regarding adjusting immunization schedules or using new adjuvants to develop more effective subunit/inactivated SARS-CoV-2 vaccines.

## Materials and Methods

### Cells and Human Serum Samples

Raji (Burkitt’s lymphoma/B lymphoblasts), THP-1 (human acute monocytic leukemia cells), and K562 (human chronic myelogenous leukemia cells) cells were cultured in Roswell Park Memorial Institute (RPMI) 1640 medium (BD, USA) supplemented with 10% v/v fetal bovine serum (FBS, Biological Industries, Israel) and 100 U/mL penicillin-streptomycin (Thermo Fisher, USA). Vero (African green monkey kidney epithelial cells) and KMB17 (human embryonic lung fibroblast-like cells) cells were cultured in Dulbecco’s modified Eagle’s medium (DMEM, BD, USA) supplemented with 10% v/v FBS and 100 U/mL penicillin-streptomycin. All cells were obtained from the Conservation Genetics Chinese Academy of Sciences Kunming Cell Bank and maintained at 37°C in an environment with 5% CO_2_ before use.

Human sera from a phase II clinical trial (Clinical trials registration number: NCT04412538) of an inactivated SARS-CoV-2 vaccine adjuvant with aluminum and SARS-CoV-2-infected human convalescent serum (HCS) were supplied by Professor Qihan Li from the Institute of Medical Biology, Chinese Academy of Medical Sciences (IMB, CAMS) (39). Specifically, healthy volunteers aged 18-59 years were intramuscularly inoculated twice with the KMS-1 SARS-CoV-2 strain (GenBank accession number MT226610.1) that was double inactivated by formaldehyde plus β-propiolactone and adjuvanted with aluminum hydroxide at medium doses (containing 100 enzyme-linked immunosorbent assay units (EUs) of viral antigen) or high doses (containing 150 EUs of viral antigen). Fourteen or 28 days after boost immunization, immune serum was collected to determine the authentic SARS-CoV-2 neutralization titers. Briefly, diluted serum samples (1:4, 1:8, 1:16, 1:32, 1:64, 1:128, and 1:256) were incubated at 37°C for 2 h with a virus at a titer 100 times higher than the 50% cell culture infectious dose (CCID50). The mixture was then added to Vero cells in 96-well plates and incubated at 37°C. After 1 week, the viral cytopathic effect (CPE) was observed and assessed with an inverted microscope (Nikon, Tokyo, Japan). The neutralization titers were defined as the highest dilution at which no CPE was observed, and neutralization titers under 4 were defined as 1 for calculation. Typical serum (N=5) with positive seroconversion, including neutralization titers equal to 4, 8, 32, 128, and 256, was collected randomly for this study.

### Immunization of Mice

Specific pathogen-free (SPF) female BALB/c mice at 6 weeks of age (14-17 g) were supplied and maintained by the Central Services of the IMB, CAMS. The animals were randomly divided into 3 groups, and each group consisted of 6 mice (N=6). SARS-CoV-2 S1 proteins expressed by HEK293 cells were purchased from Sino Biological Inc. (China). The purity of S1 was >90% as determined by SDS–PAGE and >95% as determined by size-exclusion chromatography high-performance liquid chromatography. S1 was diluted to 5 μg/mouse/dose in 25 µL of phosphate-buffered saline (PBS, pH 7.40) and mixed with the same volume of aluminum (Thermo Fisher, USA) to induce a typical Th2 response or nucleic acid immunostimulant mixtures that have been proven to induce a typical Th1 response (40). The nucleic acid immunostimulant mixtures contained 20 µg/mouse/dose oligodeoxynucleotide containing CpG motifs (CpG ODN 2395, from *In vivo*Gen, USA) and 25 µg/mouse/dose low-molecular-weight polyinosinic-polycytidylic acid (poly(I:C), from *In vivo*Gen, USA). Fifty microliters of immunogens or PBS (sham group) were administered intramuscularly to the thigh muscle three times at 2-week intervals.

### Enzyme-Linked Immunosorbent Assay (ELISA) of Antibody Titers

Two weeks after the final immunization, mice were anesthetized by an intraperitoneal injection of tribromoethanol, and blood was collected *via* cardiac puncture. After overnight clotting at 4°C, serum was collected by centrifugation at 1000 g for 10 min and pooled for further analysis. S1-specific IgG/IgG1/IgG2a titers were detected by ELISA (41, 42), and the IgG1-to-IgG2a titer ratio was calculated to evaluate the Th1-Th2 balance described previously (43, 44). Specifically, HEK293 cells expressing SARS-CoV-2 S1 protein (Sino Biological Inc., China) at 2 µg/mL were coated on 96-well plates overnight at 4°C. The plates were washed with wash buffer (0.05% (v/v) polysorbate 20 in PBS) once and then blocked with 5% (v/v) skim milk dissolved in wash buffer for 1 h at 37°C. The plates were washed four times and incubated with serially diluted mouse sera for 1 h at 37°C. After five washes, the plates were incubated with goat anti-mouse IgG/IgG1/IgG2a HRP-conjugated secondary antibodies (Thermo Fisher Scientific, USA) for 1 h at 37°C. After five washes, 3,3’,5,5’-tetramethylbenzidine (TMB, BD Bioscience, USA) substrate was added, the plates were incubated in the dark at room temperature for 10 min. The reactions were stopped by adding 2 M sulfuric acid, and the absorbance at 450 nm was detected using a microplate reader (Bio-Tek Instruments, Inc., USA). The antibody titers were defined by end-point dilution with a cutoff signal of OD450 = 0.1.

### Western Blot

Cells were cultured in 6-well plates until the concentration reached 1×10^6^ cells/mL for protein expression analysis. After three washes with PBS, radioimmunoprecipitation (RIPA) lysis buffer (Sigma, USA) supplemented with 1% protease inhibitor cocktail (MedChemExpress, USA) was added for the extraction of cellular protein. After quantification with a bicinchoninic acid (BCA) protein assay kit (Beyotime, China), 10 μg of total protein was subjected to sodium dodecyl sulfate-polyacrylamide gel electrophoresis (SDS–PAGE) and transferred to a polyvinylidene fluoride (PVDF) membrane. After blocking with 5% milk, antibodies (Abcam, USA) against angiotensin-converting enzyme 2 (anti-ACE2, 1:5 000), FcγR1 (anti-CD64, 1:10 000) and FcγR2 (anti-CD32A+CD32B+CD32C, 1:10 000) were used for assessing the protein expression of cells. A mouse monoclonal antibody against β-actin (Multi Sciences Biotech, China) was used to identify the quality of the protein extracted. Detection was performed using the enhanced chemiluminescence (ECL) reagent (Multi Sciences Biotech, China).

### Reverse Transcriptase PCR (RT–PCR)

Cells were cultured in 6-well plates at a concentration of 1×10^6^ cells/mL for gene transcription analysis. Total RNA of cells was isolated with TRIzol™ Reagent (Thermo Fisher Scientific, USA) and stored at -80°C until use. According to the manufacturer’s instructions, cDNAs were constructed with a PrimeScript™ RT Reagent Kit with gDNA Eraser (Takara, China). Briefly, 2 μL of 5×gDNA Eraser Buffer, 1 μL of gDNA Eraser, and 7 μL of RNA dissolved in RNase-free water were mixed to obtain a total volume of 10 μL. After incubation at 42°C for 2 min, the mixture was then added to the reaction solution, which contained 1 μL of PrimeScript RT Enzyme Mix I, 1 μL of RT Primer Mix, 4 μL of 5× PrimeScript Buffer, and 4 μL of RNase-free water. cDNAs were synthesized using the following PCR procedure: 37°C for 15 min, 85°C for 5 s, and maintained at 4°C.

RT–PCR was performed using previously described primer pairs to examine the expression of the genes encoding human ACE2, human FcγR1A, human FcγR2A, human FcγR2B, and human glyceraldehyde-3-phosphate dehydrogenase (GAPDH) (5). The PCRs were initialized using a general procedure of 94°C for 5 min, 30 cycles of denaturation at 94°C for 45 s, annealing at 60°C for 45 s, and extension at 72°C for 45 s, and a final extension step of 72°C for 7 min. The PCR products were verified by 2% agarose gel electrophoresis.

### Pseudovirus-Based Neutralization Assay

SARS-CoV-2 S pseudotyped virus was used for neutralization assays in biosafety level 2 facilities. This pseudovirus was based on vesicular stomatitis virus (VSV) with the G gene replaced by the firefly luciferase reporter gene and the spike protein from SARS-CoV-2 incorporated as the membrane protein (45). Specifically, the pseudoviruses were diluted to 1.3×10^4^ 50% tissue culture infectious dose (TCID50)/mL with complete DMEM before use. Fifty microliters of diluted SARS-CoV-2 S pseudovirus and 50 μL of immune serum in serial dilutions were coincubated at 37°C in an environment with 5% CO_2_ for 60 min. Subsequently, 100 μL of Vero-E6 cells (2×10^5^ cells/mL) was seeded in each mixture for another 24 h at 37°C in an atmosphere with 5% CO_2_. Finally, 100 μL of supernatant was discarded before testing. A cell control (CC), in which only cells with culture medium were added, and a virus control (VC), in which only pseudovirus and cells but no serum were added, were established separately. According to the manual protocol, the luciferase activity was assayed using a Britelite Plus Ultra-High Sensitivity Luminescence Reporter Gene Assay System (PerkinElmer, USA) and monitored using an EnSight™ Multimode Plate Reader (PerkinElmer, USA) (45).

### Pseudovirus-Based Antibody-Dependent Enhancement (ADE) of Infection *In Vitro*

The ADE of infection was evaluated *in vitro* using immune cell lines with different FcR expression patterns. Briefly, 25 μL of serially diluted serum and 25 μL of SARS-CoV-2 S pseudovirus (containing 250 TCID50 pseudoviruses) were incubated at 37°C in an atmosphere with 5% CO_2_ for 60 min. Then, 100 μL of cells (2×10^5^ cells/mL) was added to the mixtures for an additional 24 h of incubation. Afterward, the plates were centrifuged for 10 min at 300 ×g, and 50 μL of cell supernatant was discarded before testing. Cell control (CC), virus control (VC), and luciferase activity assays were performed as described above (37).

### Data Analysis

The data were processed with GraphPad Prism 7.0 (San Diego, CA, USA) and are shown as the means and standard deviations.

## Results

### In SARS-CoV-2 S1 Subunit Vaccine-Immunized Mice, Aluminum Induces Th2 Responses, and Nucleic Acid Adjuvants Induce Th1 Responses

**Figure 1:**
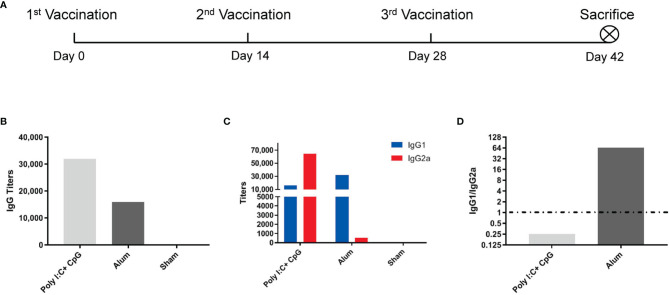
Alum induced a Th2-oriented immune response, whereas nucleic acids induced a Th1-oriented response when adjuvanted with the S1 protein. **(A)** Immunization schedule, N=6 in each group; **(B)** S1-specific IgG titers of immune serum; **(C)** S1-specific IgG1 and IgG2a titers in immune serum; **(D)** IgG1/IgG2a ratio of immune serum. All of the above titers were detected in pooled serum from each immunized group, and the means and standard deviations of duplicates are shown.

Two weeks after the third immunization (**Figure 1A**), vaccines with nucleic acid adjuvants (i.e., 20 μg of CpG ODN 2395 + 25 μg of poly(I:C)/mouse/dose) induced total S1-specific IgG antibody titers that were twofold higher (32,000 compared with 16,000) than those obtained with alum adjuvants (**Figure 1B**) and substantially higher production of S1-specific IgG2a subtype antibody (64,000 versus 500) (**Figure 1C**). Although the S1-specific IgG1/IgG2a ratio in nucleic acid-adjuvanted immunized serum was 0.25, the IgG1/IgG2a ratio in alum-adjuvanted immunized serum was as high as 64, which is a typical Th2-oriented immune response (**Figure 1D**).

### Certification of Receptor Expression

**Figure 2:**
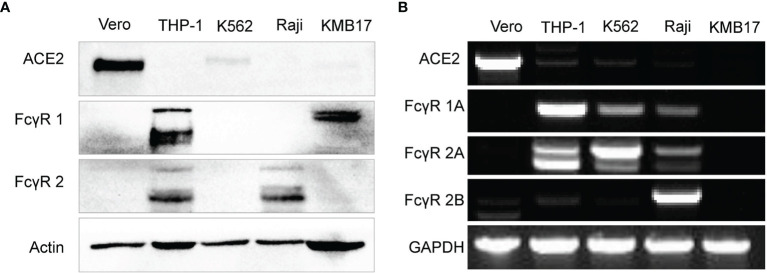
Fc receptor expression patterns in immune cell lines. **(A)** The protein expression levels of ACE2, FcγR1 and FcγR2 were detected by Western blot. **(B)** The gene expression of ACE2 and more specific FcγR subtypes, including FcγR1A, FcγR2A, and FcγR2B, was detected by RT-PCR.

Both expressed proteins (**Figure 2A**) and transcribed genes (**Figure 2B**) of ACE2 (the main receptor for SARS-CoV-2 infection) but not any type of FcγR were detected in Vero cells that were used for the proliferation of SARS-CoV-2 for inactivated vaccines, which makes Vero cells suitable for detection of the FcγR-independent enhancement of infection by serum (4, 46).

The protein level of FcγR1 in THP-1 and KMB17 cells was detected (**Figure 2A**). According to a detailed subtype analysis at the gene transcription level (**Figure 2B**), FcγR1A was expressed in THP-1 cells, and the other type of FcγR1, i.e., FcγR1B, was expressed in KMB17 cells, possibly as a pseudogene.

FcγR2 was detected at the protein level in Raji and THP-1 cells (**Figure 2A**). According to a detailed subtype analysis at the transcribed gene level (**Figure 2B**), FcγR2B was expressed in Raji cells, and FcγR2A was expressed in THP-1 cells. Although the gene transcription of FcγR2A was also detected in K562 cells, a Western blot assay indicated that the corresponding protein was not expressed (**Figure 2A**).

### Th2-Oriented Immune Serum From S1-Based Subunit Vaccines Does Not Enhance Infection *In Vitro*

**Figure 3:**
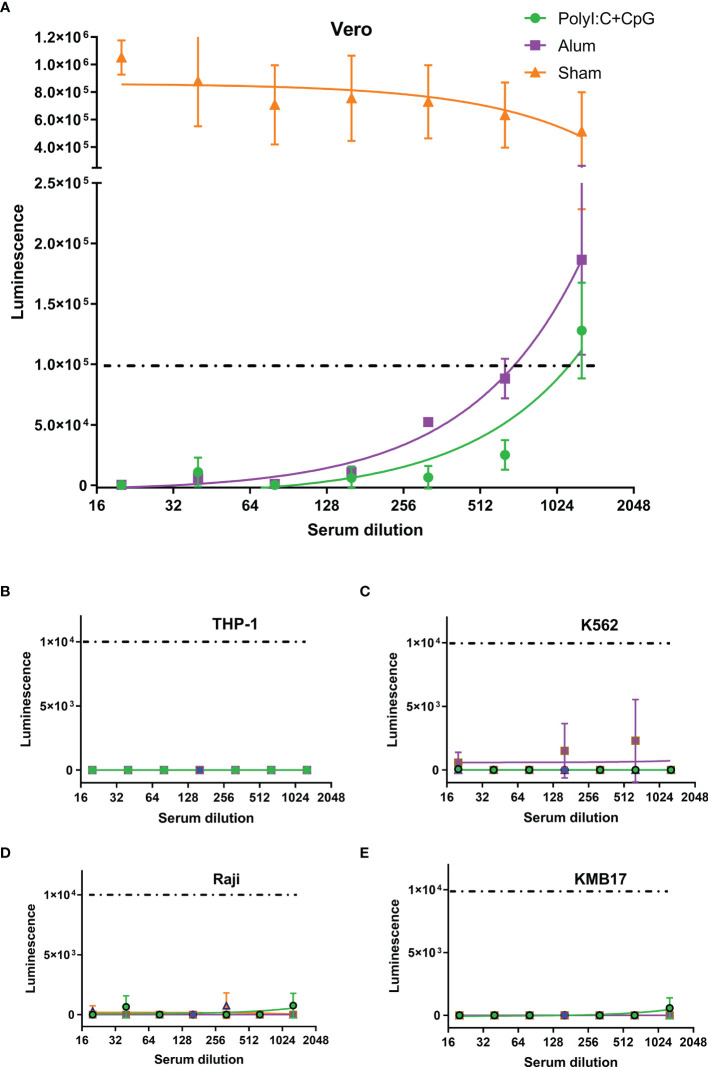
Th2-oriented immune serum after S1-based subunit vaccine administration does not enhance infection *in vitro*. **(A)** Pseudovirus-based neutralization assay of immune serum in Vero cells that express ACE2. **(B–E)** Assay of pseudovirus-based antibody-dependent enhancement of infection with immune serum in different FcγR-expressing cells. Poly(I:C)+CpG, nucleic acid-adjuvanted plus S1-purified protein immune serum; Alum, alum-adjuvanted plus S1-purified protein immune serum; Sham, serum from mice intramuscularly administered PBS instead of immunogens. All of the above analyses were performed using pooled serum from each immunized group. The means and standard deviations of duplicates are shown.

For the pseudovirus-based neutralization assay, although no luminescence was detected in the Vero cell control (CC), the luminescence of the virus control (VC) group was as high as 6×10^5^, which implied successful infection of Vero cells by SARS-CoV-2 S pseudotyped virus. Although the serum of PBS-administered mice (Sham in **Figure 3A**) showed no influence on pseudovirus infection, serum from S1-immunized mice blocked pseudovirus entry. If a luminescence of 1×10^5^ was taken as the cutoff value (which represents a more than 80% reduction in luminescence compared with that of the VC control), the virus neutralization titer (VNT) for serum from the nucleic acid-adjuvanted S1 subunit vaccine group was approximately 1150, which was higher than that for serum from the alum-adjuvanted S1 subunit vaccine group (VNT=700).

In the *in vitro* assays of the pseudovirus-based ADE of infection, even luminescence as low as 1×10^4^ (1/10 of that in the pseudovirus-based neutralization assay in **Figure 3A**, and background values around those detected in the VC control groups for each of the four cells) was taken as the cutoff value, and serum from neither the nucleic acid-adjuvanted S1 subunit vaccine group nor the alum-adjuvanted S1 subunit vaccine group showed ADE of infection in any of the four tested cell lines (**Figures 3B–E**). These cell lines included Raji cells that were confirmed to exhibit FcγR2B expression, which contributes to the enhancement of SARS-CoV-2 infection (37, 46), and THP-1 cells were confirmed to exhibit FcγR2A expression, which contributes to enhancement of MERS-CoV (47) and SARS-CoV-1 (5, 48) infection.

### Th2-Oriented Immune Serum After SARS-CoV-2 Inactivated Vaccine Administration Does Not Enhance Infection *In Vitro*

Considering that differences exist between the Fc fragment of mouse IgG and human IgG and that antibodies targeting other parts of the SARS-CoV-2 antigen except for the S1 segment may also show potential to promote infection, human sera after an inactivated SARS-CoV-2 vaccine adjuvant with alum were also tested in the abovementioned cell lines.

**Figure 4:**
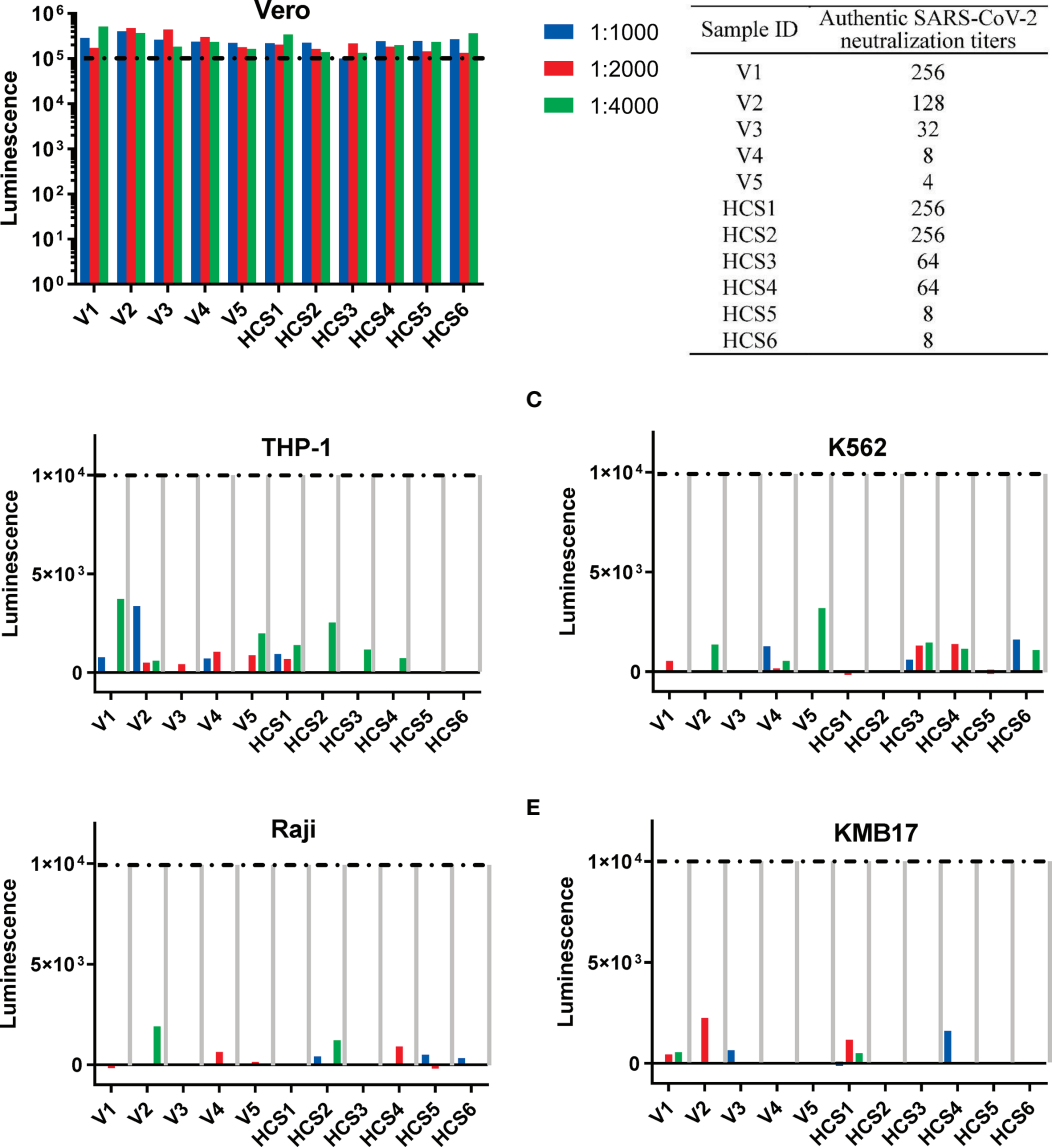
Th2-oriented immune serum after SARS-CoV-2 inactivated vaccine administration does not enhance infection *in vitro*. **(A)** Pseudovirus-based neutralization assay of human serum with low to high authentic SARS-CoV-2 neutralization titers (from 4 to 256) in Vero cells that express ACE2. The sample ID and authentic SARS-CoV-2 neutralization titer are shown in the table on the right. **(B–E)** Assay of pseudovirus-based antibody-dependent enhancement of infection with human serum in cells with different FcγR expression patterns. V1-V5, human sera after inactivated SARS-CoV-2 vaccination; HSC1-6: human convalescent serum. Each sample is shown separately.

For the pseudovirus-based neutralization assay (**Figure 4A**), none of the five sera from the SARS-CoV-2 inactivated vaccine groups, nor the six HCSs efficiently blocked the entrance of pseudovirus at a dilution of 1:1000, as reflected by corresponding luminescence reads that were higher than the cutoff value of 1×10^5^ established in the previous section (**Figure 3A**).

In the *in vitro* assay of the pseudovirus-based ADE of infection, none of the five sera from the SARS-CoV-2 inactivated vaccine groups, nor the six HCSs showed ADE of infection (shown as luminescence lower than 1×10^4^, the cutoff value for ADE discussed in the above subsection) in all four cell lines with serum dilutions from 1:1000 to 1:4000 (**Figures 4B–E**), as previously reported for the dilutions to detect ADE of infection with the SARS-CoV-1 subunit and inactivated vaccines adjuvanted with alum (5).

## Discussion

According to preclinical studies of MERS-CoV and SARS-CoV-1 vaccines, both alum-adjuvanted spike-protein-based subunits and inactivated vaccines provide partial protection upon challenge, which means that although the viral loads are lower than those in infected control animals, pulmonary immunopathology has been observed in both murine and NHP models (1–4). Notably, these immunopathologies were exacerbated compared with those of the infected control animals and were thus designated VAERD. An *in vitro* analysis showed that sera from these vaccines could enhance the infection of viruses in immune cell lines with different patterns of FcRs (5, 6). Concurrent with the reduced viral loads observed *in vivo*, these *in vitro* infections are abortive, but eliminating the virus is associated with releasing multiple proinflammatory cytokines that may cause a pulmonary immune pathology *in vivo* (5, 7–10).

Mouse serum resulting from three administrations of S1 protein adjuvanted with nucleic acid adjuvants induced higher SARS-CoV-2 neutralization titers than those in human serum after two administrations of inactivated SARS-CoV-2 vaccines, as reflected by the results showing that mouse serum resulting from the administration of S1 protein adjuvanted with nucleic acid adjuvants could still efficiently block the entrance of pseudovirus at a dilution of 1:1150 (**Figure 3A**), whereas none of the sera from alum-adjuvanted inactivated SARS-CoV-2 vaccines could block the entrance of pseudovirus at a dilution of 1:1000 (**Figure 4A**). The extent to which formaldehyde treatment alters the native conformations of viral immunogens and may therefore affect the humoral immune responses elicited by inactivated vaccines remains to be further investigated.

Consistent with previous reports on anti-SARS-CoV-1 antibodies in which none of the sera-inducing ADE of infection contain IgG2a antibodies (5), recent reports suggest that the SARS-CoV-2-targeting antibodies inducing ADE of infection are all IgG1 subtype monoclonal antibodies (36–38). Compared with immune sera from SARS-CoV-1 and MERS, which mainly depend on FcγR1 and FcγR2a for ADE of infection, ADE of infection by monoclonal antibodies against SARS-CoV-2 could be independent of FcγR (i.e., ACE2-dependent) or dependent on FcγR2b or FcγR1 (5, 37, 46). Nonetheless, after administering either alum- or nucleic acid-adjuvanted spike protein, immune serum contained polyclonal antibodies of both the IgG1 and IgG2a subtypes, although at different proportions (**Figure 1**). None of these polyclonal antibodies induced either ACE2-dependent or FcγR-dependent enhancement of infection, even at subneutralizing or non-neutralizing concentrations (**Figure 3**). A similar conclusion could also be drawn for human serum after inactivated SARS-CoV-2 vaccines that contain full-length spike proteins and other antigens, e.g., nucleocapsid proteins (5, 49) (**Figure 4**).

These results again turned our attention to Th2-type immunopathology. Previous preclinical research on both spike protein-based SARS-CoV-1 subunit vaccines and inactivated vaccines have shown that formulations with Th1-oriented adjuvants, including delta inulin and Toll-like receptor agonists, could prevent or reduce excess eosinophilic infiltration in the lungs, alleviate pulmonary eosinophilic immunopathology and enhance vaccine protection in mouse models (12, 13). These SARS-CoV-1 vaccine preclinical studies are consistent with SARS-CoV-2 phase III clinical trials due to the higher protection rate of Th1-oriented mRNA vaccines and M-matrix-adjuvanted spike protein-based subunit vaccines (50–52). Indeed, trace amounts of virus that vaccinated people primarily encounter could be eliminated more easily than the high dose of viruses administered to animal models, and this finding stresses the influence of the immune pathology on the comparably lower protection rate of inactivated SARS-CoV-2 vaccines adjuvanted with alum, which induced typical Th2-oriented immune responses (24–27, 53). Although no ERD typical of an increased eosinophilic proinflammatory pulmonary response upon challenge has been detected in preclinical studies of these inactivated SARS-CoV-2 vaccines in both murine and NHP pneumonia models (28–30), a recent parallel preclinical study of a SARS-CoV-2 mRNA vaccine and a full-length spike protein subunit vaccine adjuvanted with alum showed VAERD in mouse pneumonia models for the later (54). This result appears to be more reasonable and consistent with the conclusions from previous SARS-CoV-1 and MERS-CoV studies.

In conclusion, our studies demonstrated that Th2-oriented immune serum after SARS-CoV-2 vaccination does not enhance infection *in vitro*. We infer that the lower protection rate of inactivated SARS-CoV-2 vaccines may result from lower or waning induction of neutralization antibody production or a pulmonary eosinophilic immunopathology accompanied by eosinophilic infiltration in the lungs upon virus exposure. The immunization schedule and new adjuvants that can elevate neutralizing antibody levels and induce Th1-oriented responses to avoid a potential pulmonary eosinophilic immunopathology may be considered to elevate the protection rate of both inactivated and spike protein-based subunit SARS-CoV-2 vaccines (55–58).

## Data Availability Statement

The raw data supporting the conclusions of this article will be made available by the authors, without undue reservation.

## Ethics Statement

The studies involving human participants were reviewed and approved by the Experimental Management Association of the IMB, CAMS. The patients/participants provided their written informed consent to participate in this study. The animal study was reviewed and approved by the Ethics Committee of Animal Care and Welfare of IMB, CAMS, and the Yunnan Provincial Experimental Animal Management Association.

## Author Contributions

Conceptualization, CL. Data curation, NL and TL. Investigation, NL, TL, YW, HC, XY, and KL. Project administration, CL. Supervision, CL. All the authors have read and agreed to the published version of the manuscript.

## Funding

This work was supported by the National Key R&D Program of China (grant numbers 2020YFC0849700 and 2020YFC0860600), the Major Science and Technology Special Projects of Yunnan Province (grant numbers 202003AC100009 and 202002AA100009), the CAMS Innovation Fund for Medical Sciences (CIFMS) (grant number 2021-I2M-1-043), the Nonprofit Central Research Institute Fund of the Chinese Academy of Medical Sciences (2021-JKCS-012), the Special Biomedicine Projects of Yunnan Province (202102AA310035), the National Natural Science Foundation of China (82104130), the Fundamental Research Funds for the Central Universities (3332021072), the Basic Research Projects of Yunnan Province (202101AU070176 and 202101AT070286), the Funds for the Training of High-Level Health Technical Personnel in Yunnan Province (grant number H-2019063) and the Funds for High-level Scientific and Technological Talents Selection Special Project of Yunnan Province.

## Conflict of Interest

The authors declare that the research was conducted in the absence of any commercial or financial relationships that could be construed as a potential conflict of interest.

## Publisher’s Note

All claims expressed in this article are solely those of the authors and do not necessarily represent those of their affiliated organizations, or those of the publisher, the editors and the reviewers. Any product that may be evaluated in this article, or claim that may be made by its manufacturer, is not guaranteed or endorsed by the publisher.
